# Clinical Evaluation of an Innovative Metal-Artifact-Reduction Algorithm in FD-CT Angiography in Cerebral Aneurysms Treated by Endovascular Coiling or Surgical Clipping

**DOI:** 10.3390/diagnostics12051140

**Published:** 2022-05-04

**Authors:** Felix Eisenhut, Manuel Alexander Schmidt, Alexander Kalik, Tobias Struffert, Julian Feulner, Sven-Martin Schlaffer, Michael Manhart, Arnd Doerfler, Stefan Lang

**Affiliations:** 1 Department of Neuroradiology, University Hospital Erlangen, Friedrich-Alexander University Erlangen-Nuremberg, Schwabachanlage 6, 91054 Erlangen, Germany; manuel.schmidt@uk-erlangen.de (M.A.S.); alexander.kalik@rdtm.de (A.K.); tobias.struffert@uk-gm.de (T.S.); arnd.doerfler@uk-erlangen.de (A.D.); s.lang@uk-erlangen.de (S.L.); 2 Department of Radiology, Unfallkrankenhaus Berlin, Warener Straße 7, 12683 Berlin, Germany; 3 Department of Neuroradiology, University Hospital Giessen, Klinikstr 33, 35392 Giessen, Germany; 4 Department of Neurosurgery, University Hospital Erlangen, Schwabachanlage 6, 91054 Erlangen, Germany; julian.feulner@uk-erlangen.de (J.F.); sven.schlaffer@uk-erlangen.de (S.-M.S.); 5 Advanced Therapies, Innovation, Siemens Healthcare GmbH, Siemensstraße 1, 91301 Forchheim, Germany; michael.manhart@siemens-healthineers.com

**Keywords:** cerebral aneurysm, endovascular coiling, flat-detector computed tomography angiography, metal-artifact-reduction algorithm, surgical clipping

## Abstract

Treated cerebral aneurysms (IA) require follow-up imaging to ensure occlusion. Metal artifacts complicate radiologic assessment. Our aim was to evaluate an innovative metal-artifact-reduction (iMAR) algorithm for flat-detector computed tomography angiography (FD-CTA) regarding image quality (IQ) and detection of aneurysm residua/reperfusion in comparison to 2D digital subtraction angiography (DSA). Patients with IAs treated by endovascular coiling or clipping underwent both FD-CTA and DSA. FD-CTA datasets were postprocessed with/without iMAR algorithm (MAR+/MAR−). Evaluation of all FD-CTA and DSA datasets regarding qualitative (IQ, MAR) and quantitative (coil package diameter/CPD) parameters was performed. Aneurysm occlusion was assessed for each dataset and compared to DSA findings. In total, 40 IAs were analyzed (n_coiling_ = 24; n_clipping_ = 16). All iMAR+ datasets demonstrated significantly better IQ (*p*_IQ_ _coiling_ < 0.0001; *p*_IQ_ _clipping_ < 0.0001). iMAR significantly reduced the metal-artifact burden but did not affect the CPD. iMAR significantly improved the detection of aneurysm residua/reperfusion with excellent agreement with DSA (n_aneurysm_ _detection_ _MAR+/MAR−/DSA_ = 22/1/26). The iMAR algorithm significantly improves IQ by effective reduction of metal artifacts in FD-CTA datasets. The proposed algorithm enables reliable detection of aneurysm residua/reperfusion with good agreement to DSA. Thus, iMAR can help to reduce the need for invasive follow-up in treated IAs.

## 1. Introduction

With a prevalence of up to 6.0% [1] and an average risk for rupture of up to 6% per year [2], rupture of intracranial aneurysms (IAs) with subarachnoid hemorrhage (SAH) is associated with high morbidity and mortality and commonly has negative long term implications for survivor’s cognitive impairment, daily functionality, and quality of life [3]. Because of a potential re-bleeding in up to 15% of cases in the first few hours after initial rupture with a consequently very poor prognosis [4], prompt occlusion of ruptured IAs is absolutely indicated [5]. In unruptured IAs, treatment (including surgical clipping, endovascular coiling/stenting or watchful waiting) depends on aneurysm size, location, a patient history of previous ruptured aneurysms, family history of ruptured aneurysms, life expectancy, and growth tendencies of the aneurysm [6].

Because of its high spatial and temporal resolution, 2D digital subtraction angiography (DSA) is still the gold standard for initial detection and diagnosis as well as for follow-up control of IAs [7]. Yet, thanks to its non-invasiveness in combination with an excellent spatial resolution [8], flat-detector computed tomography angiography (FD-CTA) became a valuable alternative to DSA in post-treatment and long-term monitoring of IAs [9], especially with respect to reported post-therapeutical reperfusion rates of IAs of 2.9% up to 33.6% [10,11]. However, FD-CTA of treated IAs is limited by coil- or clip-associated metal artifacts. At worst, these artifacts lead to completely non-assessable datasets. To meet this challenge, several metal-artifacts-reduction (MAR) algorithms have been developed not only for FD-CTA [12,13,14,15], but also, for example, for mobile cone beam CT imaging of the spine [16], for CT imaging in patients with head and neck cancer and dental implants [17], or patients with suspected bladder malignancy and hip prostheses [18], and even patients with complex lower extremity fractures and external fixators [19]. In all examples, the MAR algorithms demonstrated significant artifact reduction with improved diagnostic quality. However, initial MAR algorithms did not allow the neuroradiological assessment of vascular details close to the implants and sometimes even added additional artifacts around the treated IAs [20].

**Figure 1:**
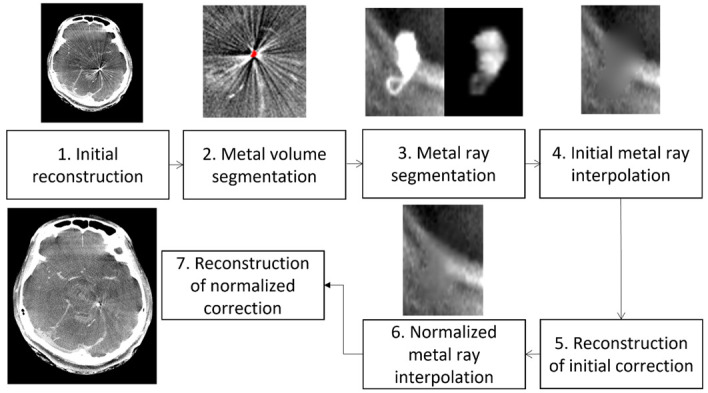
Flowchart of the iMAR technique. The interpolation-based iMAR technique is described in detail by Meyer et al. [21,22].

In this context, a novel generation of innovative MAR (iMAR) algorithm for FD-CTA was introduced by Meyer et al. using normalized metal artifact reduction, i.e., an inpainting-based MAR where the projection images are normalized using a reprojection of a prior image generated from an initial reconstruction before interpolation to improve the inpainting result. Figure 1 shows a flowchart demonstrating the iMAR postprocessing [21]. From a technical view, this algorithm provides a broad range of applications and is computationally inexpensive. Thus, the authors concluded that the iMAR algorithm might allow an effective use in clinical routine [22].

Subsequently, Amelung et al. were the first to evaluate this algorithm in clinical cases: in 21 patients with miscellaneously treated IAs (coiling, stent-assisted coiling, flow-diverter, surgical clipping, and different combinations of these methods, e.g., clipping plus flow-diverter), several different FD-CTA protocols (5s rotational angiography, 10s and 20s intravenous FD-CTA) were assessed regarding the diagnostic value of iMAR. The authors describe promising results of iMAR application providing sufficient metal artifact reduction with consecutive improvement of image quality [23].

Motivated by these findings, our aim was to validate previous results by testing such a normalized MAR algorithm in a larger patient cohort with a focus on IAs treated via coiling or clipping. Furthermore, we wanted to compare the diagnostic information of iMAR reconstructions to DSA regarding aneurysm occlusion.

Therefore, we recruited patients with treated intracranial aneurysms, postprocessed the acquired datasets, and assessed the postinterventional DSA and FD-CTA images regarding image quality and metal artifacts, compared iMAR+ and iMAR− FD-CTA images and summarize and discuss our results with the findings of other previous studies evaluating metal-artifact-reduction algorithms.

## 2. Materials and Methods

### 2.1. Patients

Patients with IAs treated either via endovascular coiling using standard coils (CERENOVUS, Irvine, CA, USA) or surgical clipping using standard titanium clips (MIZUHO Corporation, Tokyo, Japan; B. Braun Deutschland GmbH & Co. KG, Tuttlingen, Germany) from January 2015 to December 2019 were considered for our analysis with a special focus on patients with IA reperfusion/residuum. In particular, exclusively patients with complete in-house follow-up comprising both DSA and FD-CTA 6 months after IA treatment were considered.

Written informed consent for DSA and FD-CTA was obtained from all patients. The study was performed according to the Declaration of Helsinki and the European Guidelines for Good Clinical Practice. Additional ethical review was not required for participation in this retrospective analysis in accordance with local legislation (BayKrG Section 27, paragraph 4) and institutional requirements.

### 2.2. Acquisition and Postprocessing

#### 2.2.1. FD-CTA

FD-CTA was performed on a biplane angiography system (Axiom Artis dBA, Siemens Healthcare GmbH, Erlangen, Germany) using a standard protocol (10sDSA, Siemens Healthcare GmbH, Erlangen, Germany) with the following parameters: scan time 2 × 10 s, 200° total rotation angle, 0.8°/frame, about 250 frames total, matrix 512 × 512, 70 kV, 1.2 μGy/frame. As previously described [24,25], the bolus-watching technique for best arterial contrast was applied. Using a power injector (Accutron HP-D, Medtron, Saarbrücken, Germany), 60 mL contrast agent (Imeron 400, Bracco Imaging, Konstanz, Germany) followed by a 60 mL saline flush was intravenously injected with a flow-rate of 5 mL/s.

#### 2.2.2. DSA

DSA was performed on a biplane angiography system (Axiom Artis dBA, Siemens Healthcare GmbH, Erlangen, Germany) using a diagnostic catheter on a standard transfemoral route. Posteroanterior, lateral, and oblique 2D projections and a 3D rotational angiography of the IAs were acquired using a standard protocol (5s-DSA, Siemens Healthcare GmbH, Erlangen, Germany) with manual injection of 10 mL contrast agent (Imeron 400, Bracco Imaging, Konstanz, Germany).

#### 2.2.3. Postprocessing

The raw data of the FD-CTA and the 3D rotational angiography was postprocessed on a dedicated workstation (syngo X Workplace, Siemens Healthcare GmbH, Erlangen, Germany) running commercially available software (syngo DynaCT SMART, Siemens Healthcare GmbH, Erlangen, Germany).

For each FD-CTA run, two datasets were postprocessed with/without application of the iMAR algorithm (iMAR+/iMAR−) as described by Meyer et al. [22]. Standard reconstruction of all iMAR−/iMAR+ datasets was performed with the following parameters: kernel type ‘HU’, image impression ‘normal’, mode ‘NatFill’, matrix 512 × 512, voxel size < 0.15 mm. Standard postprocessing of 3D rotational angiography was performed with the following parameters: kernel type ‘EE’, image impression ‘smooth’, mode ‘subtraction with motion correction’, matrix 512 × 512, voxel size < 0.15 mm. Then, triplanar maximum intensity projections (MIPs) aligned to the IA were reconstructed with a slice thickness and slice distance of 0.5 mm.

**Figure 2:**
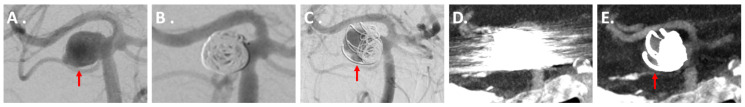
A 63-year-old patient with an aneurysm of the basilar artery in the initial DSA ((**A**) red arrow) with consecutive treatment via endovascular coiling (**B**). Six months later, DSA control revealed a reperfusion in the aneurysm center ((**C**) red arrow). The aneurysm was not assessable in iMAR− FD-CTA due to severe metal artifacts (**D**). After application of iMAR and the following significant metal artifact reduction, FD-CTA also demonstrated reperfusion in the aneurysm center ((**E**) red arrow).

**Figure 3:**
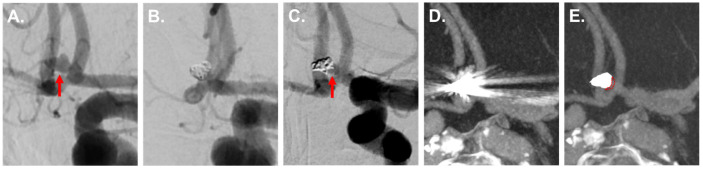
An 84-year-old patient with an aneurysm of the anterior communicating artery in the initial DSA ((**A**) red arrow) with consecutive treatment via endovascular coiling (**B**). Six months later, DSA control revealed a reperfusion at the aneurysm base ((**C**) red arrow). The aneurysm was not assessable in iMAR− FD-CTA due to severe metal artifacts (**D**). Thanks to a significant metal artifact reduction, iMAR+ FD-CTA also demonstrated the basal aneurysm reperfusion in accordance with the DSA ((**E**) marked with red dotted line).

**Figure 4:**
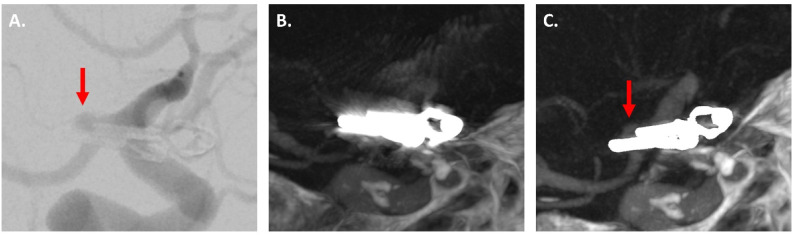
An 80-year-old patient with an aneurysm of the right posterior communicating artery treated via intracranial clipping. DSA control revealed a small residuum at the aneurysm neck ((**A**) red arrow). The aneurysm was not assessable in iMAR− FD-CTA due to severe metal artifacts (**B**). After application of iMAR and the following significant metal artifact reduction, FD-CTA also demonstrated the small residuum ((**C**) red arrow).

Figure 2, Figure 3 and Figure 4 show exemplary images of DSA data and the corresponding iMAR−/iMAR+ FD-CTA of both patients with IAs treated via endovascular coiling and intracranial clipping.

### 2.3. Data Evaluation

All datasets were analyzed with commercially available viewing software (syngo.plaza, Siemens Healthcare GmbH, Erlangen, Germany) in consensus reading by two experienced neuroradiologists (F.E and S.L.).

#### 2.3.1. Image Quality

Image quality (IQ) regarding diagnostic value of all DSA and FD-CTA datasets was evaluated by using a 4-folded Likert scale: (4) structures perfectly distinguishable, good quality; (3) structures well distinguishable, diagnostic quality; (2) structures hardly distinguishable, poor quality; (1) structures not distinguishable, no diagnostic value.

IQ regarding metal-artifact burden of FD-CTA datasets was evaluated by using a 5-folded Likert scale: (0) no artifacts; (1) minimal artifacts; (2) moderate artifacts, parent vessel well differentiable; (3) severe artifacts, parent vessel poorly differentiable; (4) no diagnostic value, massive artifacts, parent vessel not differentiable.

**Figure 5:**
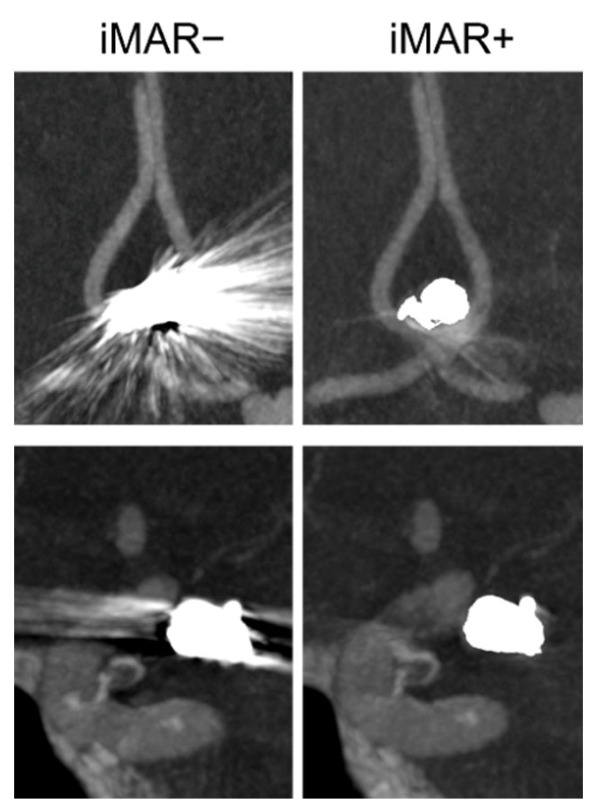
Side-by-side examples of FD-CTA images without/with application of the iMAR (iMAR−/iMAR+) in patients with treated intracranial aneurysms via endovascular coiling. Upper row shows a patient with a treated aneurysm of the anterior communicating artery, basal row shows a patient with a treated aneurysm of the internal carotid artery. In the iMAR− images of both cases, the parent vessels are not (upper case) or poorly differentiable (basal case), whereas in the iMAR+ images, the parent vessels are very well differentiable.

Figure 5 illustrates side-by-side examples of iMAR+/− FD-CTA images regarding the differentiation of the parent vessel.

#### 2.3.2. Aneurysm Occlusion

IAs treated via endovascular coiling were graded by using the modified Raymond–Roy occlusion classification (MRROC) as described by Mascitelli et al. [26]: (0) not assessable; (1) no reperfusion; (2) reperfusion at the aneurysm base; (3) reperfusion at the center of the aneurysm; (4) reperfusion along the aneurysm wall. Table 1 summarizes the MRROC for IAs treated via endovascular coiling.

IAs treated via surgical clipping were graded by using the Sindou’s classification (SC) [27]: (0) not assessable; (1) reperfusion of <50% of the aneurysm neck; (2) reperfusion of >50% of the aneurysm neck; (3) residual lobe of a multilobulated aneurysm sac; (4) residual portion < 50% of the initial aneurysm sac; (5) residual portion > 50% of the initial aneurysm sac; (6) no residuum. Table 2 summarizes the SC for IAs treated via surgical clipping.

DSA was used as reference for both IAs treated via endovascular coiling and surgical clipping.

#### 2.3.3. Coil Package Diameter (CPD)

**Figure 6:**
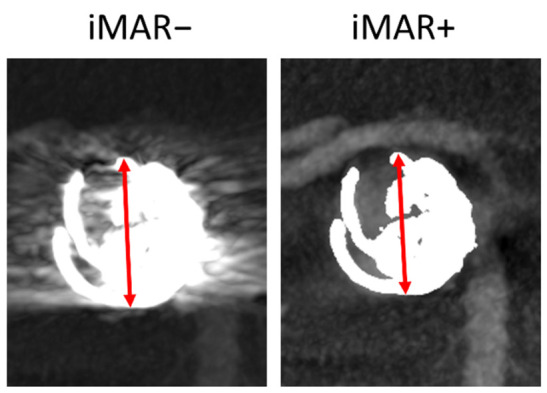
Exemplary CPD measurement in iMAR−/+ FD-CTA images. CPD is indicated by the red arrow.

In all DSA and FD-CTA datasets with IAs treated via endovascular coiling, the size of the maximum CPD (mm) was measured in the image displaying the largest coil package diameter using the standard measurement tool on the syngo X Workplace workstation. Figure 6 illustrates an exemplary CDP measurement in iMAR−/+ FD-CTA images.

### 2.4. Statistical Analysis

Image quality regarding diagnostic value and metal-artifact burden as well as CPD were analyzed by use of descriptive statistics and tested for normal distribution by using the D’Agostino-Pearson test (if *p* > 0.05, normality was accepted). Then, all values were tested for a significant difference between iMAR−/iMAR+ datasets via an unpaired, two-tailed *t*-test (if values showed a Gaussian distribution) or via the Mann–Whitney U test (if values showed no Gaussian distribution). If values of iMAR−/iMAR+ datasets demonstrated non-conforming Gaussian distributions (e.g., iMAR− values showed no Gaussian distribution and iMAR+ values showed a Gaussian distribution), the Mann–Whitney U test was used. CPD of FD-CTA were additionally compared to DSA values and tested for significant difference by using a paired, two-tailed *t*-test.

MRROC and Sindou’s grading of IAs were compared between iMAR−/iMAR+ datasets and DSA findings.

*p*-values less than 0.05 were considered statistically significant. *p*-values less than 0.05 are marked with “*”, less than 0.01 with “**”, less than 0.001 with “***”, and less than 0.0001 with “****”. For each computed *p*-value, the used statistical test (unpaired, two-tailed *t*-test (t); paired, two-tailed *t*-test (*p*); Mann–Whitney U test (u)) is indicated.

Statistical analysis was performed with GraphPad Prism 8 (GraphPad Software, San Diego, CA, USA) and Excel (Microsoft, Redmond, WA, USA).

## 3. Results

### 3.1. Patients

In total, 28 patients with treated IAs were included in our retrospective analysis: In 19 patients (n_female_ = 13; n_male_ = 6, median age = 67.9 years), 20 IAs were treated via coiling. In 6 patients (n_female_ = 5; n_male_ = 1; median age = 62.2 years), 11 IAs were treated via clipping. In 3 patients (n_female_ = 2; n_male_ = 1; median age = 62.7), IAs were treated via both coiling (n = 4) and clipping (n = 5). In total, 24 coiled IAs and 16 clipped IAs were assessed. Table 3 presents the aneurysm location and treatment method.

### 3.2. Image Quality

IQ values regarding diagnostic value showed a Gaussian distribution for iMAR+ datasets of IAs treated via coiling; the other IQ values regarding diagnostic value showed no Gaussian distribution. IQ regarding diagnostic value was rated significantly higher in iMAR+ datasets in comparison to iMAR− datasets in both coiled and clipped IAs (IQ_iMAR+ coiling_ = 3.04 ± 0.55, IQ_iMAR− coiling_ = 1.25 ± 0.53, *p*_coiling_ < 0.0001 (u); IQ_iMAR+ clipping_ = 2.81 ± 0.40, IQ_iMAR− clipping_ = 1.00 ± 0.0, *p*_clipping_ < 0.0001 (t)).

**Figure 7:**
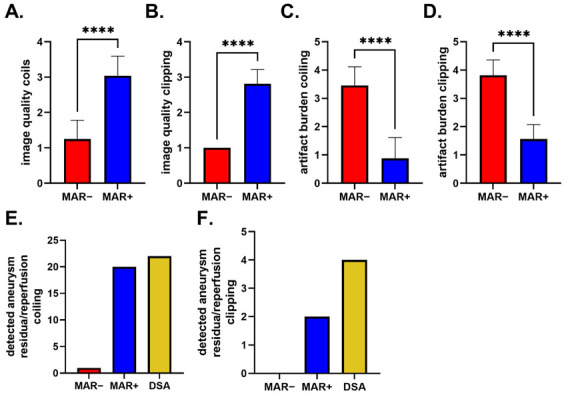
Evaluation of IQ regarding diagnostic value in patients with coiled (**A**) and clipped (**B**) IAs. The iMAR algorithm significantly improves IQ. Evaluation of IQ regarding metal-artifact burden in patients with coiled (**C**) and clipped (**D**) IAs. The iMAR algorithm significantly reduced metal-artifact burden in all cases. (**E**) Number of detected aneurysm reperfusions in patients treated via endovascular coiling. iMAR+ FD-CTA allows better aneurysm reperfusion detection with good agreement with DSA reference in comparison with iMAR− images. (**F**) Number of detected aneurysm residua in patients treated via surgical clipping. iMAR+ FD-CTA allows better aneurysm residua detection in comparison with iMAR− images. *p*-values less than 0.0001 are marked with “****”.

IQ values regarding metal-artifacts burden showed a Gaussian distribution for FD-CTA datasets of IAs treated via coiling and showed no Gaussian distribution for IAs treated via clipping. Application of the iMAR algorithm significantly reduced the metal-artifact burden in both FD-CTA datasets of coiled and clipped IAs (IQ_MA iMAR+ coiling_ = 0.88 ± 0.74, IQ_MA iMAR− coiling_= 3.46 ± 0.66, *p*_IQ MA coiling_ < 0.0001 (t); IQ_MA iMAR+ clipping_= 1.56 ± 0.51, IQ_MA iMAR− clipping_= 3.81 ± 0.54, *p*_IQ MA clipping_ < 0.0001 (u)). Table 4 and Figure 7A–D. summarize evaluation of image quality regarding diagnostic value and metal-artifact burden.

### 3.3. Aneurysm Occlusion

#### 3.3.1. IAs Treated via Coiling

In 2 DSA datasets, MRROC grading was (1); in 6 DSA datasets, grading was (2); and in 16 DSA datasets, grading was (4).

In 4 iMAR+ datasets, MRROC grading was (1); in 7 iMAR+ datasets, grading was (2); in 1 iMAR+ dataset, grading was (3); and in 12 iMAR+ datasets, grading was (4).

In 22 iMAR− datasets, MRROC grading was (0), in 1 iMAR− dataset grading was (1), and in 1 iMAR− dataset grading was (4).

Findings in iMAR+ datasets showed better agreement with the DSA reference than findings in iMAR− datasets (n_correct iMAR+_ = 19, n_correct iMAR−_ = 1). Application of the iMAR allowed the detection of 90.1% of aneurysm reperfusions (20 of 22); without the iMAR only 4.5% of aneurysm reperfusions (1 of 22) were detected.

#### 3.3.2. IAs Treated via Clipping

In 3 DSA datasets, SC grading was (1); in 1 DSA dataset, grading was (2); and in 12 DSA datasets, grading was (6).

In 1 iMAR+ dataset, SC grading was (1); in 1 iMAR+ dataset grading was (2); and in 14 iMAR+ datasets, grading was (6).

In 14 iMAR− datasets, SC grading was (0); and in 2 iMAR− datasets, grading was (6).

Findings in iMAR+ datasets showed better agreement with DSA than findings in iMAR− datasets (n_correct iMAR+_ = 14, n_correct iMAR−_= 2). Application of the iMAR allowed the detection of 50% aneurysm residua (2 of 4); without the iMAR no aneurysm residuum was detected.

Table 5 and Table 6 and Figure 7E,F summarize aneurysm occlusion grading for coiling and clipping.

### 3.4. CPD

Compared to the DSA reference, both iMAR+ and iMAR− FD-CTA datasets showed a concatenated deviation regarding coil package diameters (CPD_iMAR+_ = 6.93 ± 2.79 cm, CPD_iMAR−_ = 6.81 ± 2.71 cm, CPD_DSA_ = 6.26 ± 2.80 cm). CPD showed no significant difference between iMAR+ and iMAR− datasets (*p*_CPD iMAR+ vs_. _iMAR−_ = 0.3814 (*p*)).

## 4. Discussion

Metal artifacts are a substantial limitation of the radiologic follow-up of treated IAs—regardless of the therapeutic approach. In consequence, algorithms for metal artifact reduction have been developed to improve image quality. Here we evaluated the diagnostic value of an innovative metal-artifact-reduction algorithm [21,22] specifically designed for FD-CT applications. In particular, we focused on patients with IAs treated either via coiling or clipping—as the most susceptible cohorts for a high metal-artifact burden in their follow-up datasets. In our series, the proposed iMAR algorithm significantly improved image quality and reduced metal artifacts. Furthermore, the algorithm provided a higher detection rate of aneurysm residua and reperfusion and showed a high level of agreement with DSA as reference standard for both patient groups, respectively. As another important finding, the iMAR algorithm does not distort quantitative parameters.

As previously reported by Amelung et al., iMAR algorithms show promising results in terms of improving image quality and reduction of the metal-artifact burden. In accordance with our findings of a higher detection rate of aneurysm reperfusion/residua in iMAR+ images, application of iMAR led to a higher detection rate of aneurysm reperfusion in Amelung et al.’s series (8 of 9 cases of aneurysm reperfusion detected in MAR+ images vs. 2 of 9 cases in MAR− images). Moreover, even initially non-diagnostic datasets significantly benefitted from iMAR and became evaluable in terms of aneurysm reperfusion [23]. However, Amelung et al.’s patient cohort is characterized by a high level of heterogeneity regarding the sample size per treatment group (coiling, clipping, flow-diverter + clipping, stent-assisted coiling, clipping + coiling, clipping + stent-assisted coiling) and the applied examination protocols (iv FD-CTA 10 s, iv FD-CTA 20 s, 5 s rotational angiography). As a consequence of taking both datasets with intraarterial and intravenous contrast application into account, the vessel contrast must vary substantially in these datasets and must be seen as a relevant confounder regarding the assessment of the iMAR algorithm. In addition—as Amelung et al.’s analysis of iMAR algorithm focused on 3D rotational angiography with intraarterial contrast application (18 of 21 patients/86%)—their results are only of limited transferability to FD-CTA datasets with intravenous contrast application. However, in our experience, especially datasets with intravenously applied contrast agent regularly suffer from low vascular contrast. Hence, our detailed analysis of the iMAR algorithm for FD-CTA with intravenous contrast in a larger, homogenous patient cohort with a standardized scan protocol is another important component to assess the diagnostic value of this algorithm. In this context, we consider the high level of agreement between iMAR+ datasets with intravenous contrast and intraarterial DSA regarding aneurysm reperfusion detection rate in coiled IAs (90.1%) the most important finding of our analysis. Even in datasets of clipped IAs that suffer from significant metal-artifact burden, the iMAR algorithm allowed detection of 50% of aneurysm residua.

Moreover, there are several recent publications assessing metal-artifact-reduction algorithms for all clinically relevant imaging modalities (DSA, FD-CT, CB-CT, MS-CT, MRI, and PET-CT) [28,29,30,31,32,33]. For example, in a recent study, Zheng et al. evaluated a novel subtraction method combining metal artifact reduction and virtual monochromatic imaging to improve image quality of CT angiography in patients with endovascular coiling of intracranial aneurysms. The authors report significantly reduced coil artifacts and improved vessel visualization adjacent to the coils [34]. Furthermore, metal artifact reduction algorithms can also substantially reduce artifacts from deep brain stimulation electrodes in head CT images, as recently reported by Nagayama et al. [35]. In this context, Hakim et al. assessed an iterative metal artifact reduction algorithm in CT perfusion imaging in patients after coiling or clipping of ruptured brain aneurysms. Again, the application of their algorithm significantly improved image quality and reduced artifacts. Therefore, the authors conclude that MAR algorithms are excellent tools for reducing artifacts in CT perfusion and should be used in clinical practice [36].

Particularly with regards to neuroradiological applications of FD-CT, the first dedicated MAR algorithms were implemented and assessed in 2010: Prell et al. were the first to report on an effective algorithm reducing image noise, improving brain tissue modeling and implant visibility in native postinterventional FD-CT datasets. The authors concluded that their MAR algorithm is a promising step towards a better image quality and diagnosis in the presence of metal implants [12,37]. Taking these preliminary findings into account and refining the application of this algorithm, Psychogios et al. were the first to evaluate the diagnostic value of a MAR algorithm in FD-CT after intravenous contrast application in postinterventional cases. Despite the assessed datasets also being characterized by a small sample size (n = 16) and a high level of heterogeneity concerning the underlying pathologies (intracranial aneurysms and arterial stenosis) and the performed treatment approaches (coiling, clipping, or stenting), the authors also concluded that such a MAR algorithm significantly reduces artifacts of the metal implants and consecutively allows a sufficient delineation of adjacent structures [38]. In a similar approach but mainly focusing on clipped and (stent-assisted) coiled IAs, Stidd et al. confirmed previous results concerning the efficacy of MAR algorithms [13]. In detail, their analysis proved an optimized visualization of vascular structures adjacent to the metallic implants with a higher absolute number of delineable vessel segments. In addition, both Pjontek et al. and Yasuda et al. were able to demonstrate a significant amelioration of the visualization not only of treated aneurysms and the corresponding parent vessels but also of the brain parenchyma neighboring metal implants in MAR postprocessed FD-CTA datasets [15,20]. Thereon, Mennecke et al. could show that even a reliable detection of acute SAH is possible in postinterventional non-contrast FD-CT datasets using such a MAR algorithm [14]. These results are supported by findings of Enomoto et al. reporting improved image quality of native FD-CT in patients with intracranial aneurysms treated with coils (40 patients) and clips (four patients). The authors state that MAR software allows the precise detection of intraprocedural complications on FD-CT datasets in the intervention suite without the need for patient transfer [39]. In a recent study, Murai et al. evaluated a MAR algorithm in FD-CTA in patients with cerebral aneurysms treated with stent-assisted coil embolization. Similar to our results, the authors report improved image quality following the application of the MAR algorithm and they conclude that these algorithms could support the radiologist’s assessment of possible in-stent thrombosis or the patency of small perforating arteries [40]. In accordance, Chintalapani et al. report reduced FD-CT image noise and improved device visibility in patients with stent-assisted and flow-diverter assisted coil embolization of intracranial aneurysms after MAR algorithm application [41].

However, MAR algorithms cannot provide artifact free images in all cases. Because the visualization of implant-surrounding anatomy strongly relies on the quantity of image information of the implant-surrounding voxels, MAR algorithms might be associated with an altered illustration of implant-surrounding structures. For example, Psychogios et al. report a significant blurring of brain parenchyma and vascular structures approximately 3 mm around a clipped aneurysm [38]. In this context, Yasuda et al. observed that MAR algorithms intensify other artifact types (e.g., bone-related or implant-related artifacts) and affect soft tissue contrast [20]. Interestingly—in contrast to these observations but in accordance with Amelung et al.’s results [23]—in our series, iMAR application was not associated with decreased diagnostic information regarding brain parenchyma and parent vessel delineation. Thus, the iMAR algorithm in its current version might be more robust for reducing the metal-artifact burden and improving the visualization of the implant-surrounding anatomy in FD-CTA datatsets of patients with treated IAs. Furthermore, despite the significant improvement of overall image quality and reduction of metal artifacts, four aneurysm reperfusion/residua were not detectable in iMAR+ FD-CTA datasets in our study. In general, these aneurysm reperfusion/residua were diminutive and—as rupture risk is minimal—close follow-up imaging after detection via DSA to exclude growth is sufficient. However, especially in patients with treated intracranial aneurysms with large coil packages or after clipping and consecutive extensive metal artifacts, it is the radiologist’s responsibility to always remember the possibility of missing small aneurysm reperfusion/residua. In these cases, invasive DSA is the modality of choice to confirm sufficient aneurysm occlusion. Nevertheless, we still recommend application of iMAR in the clinical routine because of its significant image quality improvement by metal artifact reduction, its computational inexpensiveness, and most importantly its higher detection rate of aneurysm reperfusion/residua.

Our study has some limitations: first, the small number of patients with aneurysm residua after intracranial clipping; second, the retrospective analysis of the data; third, our analysis does not address stents and flow-diverters. Prospective studies with larger cohorts are needed to verify our findings.

## 5. Conclusions

The proposed iMAR algorithm provides significant metal artifact reduction and consecutive improvement of image quality in FD-CTA of patients with treated intracranial aneurysms after endovascular coiling or surgical clipping. In detail, iMAR allows reliable detection of aneurysm reperfusion following coiling with excellent agreement to DSA and also improves detection of aneurysm residua after clipping. In addition, iMAR enables reliable evaluation of the surrounding brain parenchyma and parent vessels. Most importantly, the algorithm does not distort quantitative parameters as the CPD. Therefore, this algorithm is another important component to further optimize noninvasive postinterventional diagnostics and might help to reduce the need for invasive follow-up DSA imaging.

## Figures and Tables

**Table 1:** Modified Raymond–Roy occlusion classification (MRROC) for intracerebral aneurysms treated via endovascular coiling.

MRROC	
0	not assessable
1	no reperfusion
2	reperfusion at the aneurysm base
3	reperfusion at the center of the aneurysm
4	reperfusion along the aneurysm wall

**Table 2:** Sindou’s classification (SC) for intracerebral aneurysms treated via surgical clipping.

SC	
0	not assessable
1	reperfusion of <50% of the aneurysm neck
2	reperfusion of >50% of the aneurysm neck
3	residual lobe of a multilobulated aneurysm sac
4	residual portion < 50% of the initial aneurysm sac
5	residual portion > 50% of the initial aneurysm sac
6	no residuum

**Table 3:** Aneurysm characteristics.

Treatment Method	n	
coiling	24	
clipping	16	
**aneurysm location**	**n**	
	**coiling**	**clipping**
anterior communicating artery	11	2
middle cerebral artery	4	9
basilar artery	4	/
posterior communicating artery	3	3
internal carotid artery	1	2
posterior inferior cerebellar artery	1	/

**Table 4:** Evaluation of image quality.

IQ Regarding Diagnostic Value			
	iMAR−	iMAR+	*p*-Value
coiling	1.25 ± 0.53	3.04 ± 0.55	<0.0001
clipping	1.00 ± 0.0	2.81 ± 0.40	<0.0001
**IQ regarding metal-artifacts burden**			
	**iMAR−**	**iMAR+**	***p*-Value**
coiling	3.46 ± 0.66	0.88 ± 0.74	<0.0001
clipping	3.81 ± 0.54	1.56 ± 0.51	<0.0001

IQ = image quality; iMAR−/+ = flat-panel computed tomography angiography dataset postprocessed without/with application of the iMAR algorithm.

**Table 5:** Aneurysm occlusion grading in IAs treated via coiling.

	MRROC 0	MRROC 1	MRROC 2	MRROC 3	MRROC 4	Reperfusion Detection Rate
**iMAR−**	22	1	0	0	1	1 of 22 (4.5%)
**iMAR+**	0	4	7	1	12	20 of 22 (90.1%)
**DSA**	0	2	6	0	16	22 (100%)

IA = intracerebral aneurysm; iMAR−/+ = flat-panel computed tomography angiography dataset postprocessed without/with application of the iMAR algorithm; DSA = 2D digital subtraction angiography; MRROC = modified Raymond–Roy occlusion classification.

**Table 6:** Aneurysm occlusion grading in IAs treated via clipping.

	SC 0	SC 1	SC 2	SC 3	SC 4	SC 5	SC 6	Residua Detection Rate
**iMAR−**	14	0	0	0	0	0	2	0 of 4 (0%)
**iMAR+**	0	1	1	0	0	0	14	2 of 4 (50%)
**DSA**	0	3	1	0	0	0	12	4 (100%)

IA = intracerebral aneurysm; iMAR−/+ = flat-panel computed tomography angiography dataset postprocessed without/with application of the iMAR algorithm; DSA = 2D digital subtraction angiography; SC = Sindou’s classification.

## Data Availability

The data presented in this study are available on request from the corresponding author.
